# Metabolomics and Cardiology: Toward the Path of Perinatal Programming and Personalized Medicine

**DOI:** 10.1155/2017/6970631

**Published:** 2017-07-03

**Authors:** Roberta Pintus, Pier Paolo Bassareo, Angelica Dessì, Martino Deidda, Giuseppe Mercuro, Vassilios Fanos

**Affiliations:** ^1^Department of Surgery, Neonatal Intensive Care Unit, Neonatal Pathology and Neonatal Section, University of Cagliari, Policlinico Universitario, Strada Statale 554, Km 4.500, Bivio di Sestu, Monserrato, 09042 Cagliari, Italy; ^2^Department of Medical Sciences “M. Aresu”, Unit of Cardiology and Angiology, University of Cagliari, Policlinico Universitario, Strada Statale 554, Km 4.500, Bivio di Sestu, Monserrato, 09042 Cagliari, Italy

## Abstract

Heart diseases are one of the leading causes of death in Western Countries and tend to become chronic, lowering the quality of life of the patients and ending up in a massive cost for the Health Systems and the society. Thus, there is a growing interest in finding new technologies that would allow the physician to effectively treat and prevent cardiac illnesses. Metabolomics is one of the new “omics” sciences enabling creation of a photograph of the metabolic state of an individual exposed to different environmental factors and pathologies. This review analyzed the most recent literature about this technology and its application in cardiology in order to understand the metabolic shifts that occur even before the manifestation of these pathologies to find possible early predictive biomarkers. In this way, it could be possible to find better treatments, ameliorate the patient's quality of life, and lower the death rate. This technology seems to be so promising that several industries are trying to set up kits to immediately assess the metabolites variations in order to provide a faster diagnosis and the best treatment specific for that patient, offering a further step toward the path of the development of a tailored medicine.

## 1. Introduction

Cardiac pathologies are a critical health issue affecting millions of people worldwide with a constant mortality rate in particular in the elderly, a difficult prognosis, and a worsening in quality life of affected people. In fact, they tend to become chronic and lead to several complications that may affect other vital organs such as brain, lungs, and kidneys. Indeed cardiovascular diseases (CVDs) are globally the number one cause of death: more people die every year from CVDs than from any other cause (17.5 million deaths, an estimation of 31% of all deaths worldwide). People with cardiovascular pathologies or who are at high cardiovascular risk need early detection since the 80% of premature heart diseases are preventable [1].

Among the complications of these pathologies there are pulmonary edema or respiratory tract infections, kidney insufficiency, and stroke. In children, cardiovascular diseases or congenital heart malformations can lead to pulmonary hypertension and neurodevelopmental problems due to the lack of oxygen supply [2].

The pathophysiology of heart pathologies is complex. Indeed, recent findings pointed out a possible pivotal role of mitochondrial dysfunction and the subsequent altered energy metabolism in cardiac diseases, in particular in case of heart failure [3].

In general, patient management could be quite challenging and demanding; thus there is a need for the clinician to have the best tools that can improve and facilitate the diagnosis and the prognosis for these diseases.

## 2. Metabolomics: The Skeleton Key of Cardiac Diseases?

During the last decade, animal and human studies have applied metabolomics to cardiovascular research, using both targeted and untargeted approaches; as such, metabolic fingerprints have been identified for several cardiovascular risk factors and diseases [4].

**Figure 1:**
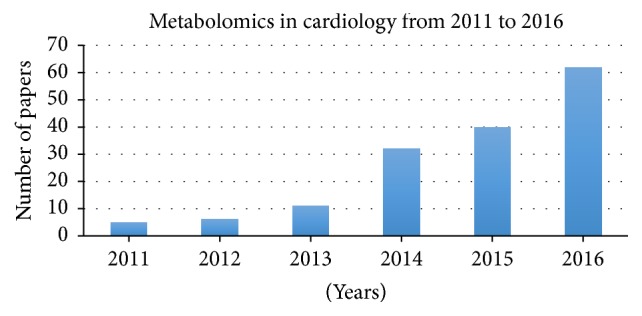
PubMed results concerning the studies of metabolomics and cardiology from 2011 to 2016.

Indeed, by entering the keywords “metabolomics” and “cardiology” on PubMed, this will show 161 papers, 156 written from 2011 to 2016 (Figure 1).

In fact metabolomics is a new technique that allows investigators to study the metabolic network involved in heart diseases so as to better understand their pathophysiological mechanism. Griffin et al. highlighted how the classical metabolomics technique could be applied in cardiology; indeed high resolution Magnetic Resonance Imaging (MRI) and mass spectrometry (MS) are extremely useful for gaining information about cardiac disease processes since they are both highly discriminant for a range of pathological processes starting from cardiac ischemia (angina and myocardial infarction) to heart failure [5]. These techniques could be applied to both heart tissue and biofluids such as blood and saliva with a minimum compliance needed from the patient, since their collection is not invasive. Metabolomics is one of the newest “omics” science and before its broad application, scientists tried to investigate the metabolic variation in both physiological and pathological states using proteomics or transcriptomics, but these techniques have several limitations; for instance, they are not “real-time” meaning that in case of disease occurrence the modification in the proteome or transcriptome modifications are much slower than modifications in the metabolome [6].

In Table 1 the most relevant studies are reported concerning metabolomics in cardiology on PubMed from 2011 to 2016. There are 18 studies, involving 3.874 patients in total: 2351 suffered from acute cardiac pathologies, while 736 suffered from chronic heart illnesses and 786 were controls. Most studies (9) performed ^1^H-NMR analysis. The prevalent biofluid analyzed was plasma (10 studies) followed by serum (7 studies), urine (3), and breath (1).

In 2011 Kang et al. investigated metabolomics urinary profiles of elderly patients with ischemic heart failure, using ^1^H-nuclear magnetic resonance (^1^H-NMR) [23]. The patients compared to controls showed different levels of ketonic bodies as a marker of altered myocardial metabolism meaning that one of the pathological features of this pathology could be a reduction in fatty acids oxidation and an increase of glucose metabolism.

Among others, the study performed by Desmoulin et al. in 2013 underlines the predictive power of metabolomics [19]. It is a prospective study on a cohort of acute heart failure patients admitted in the cardiac intensive care unit and it assessed survival at 30 days. The plasma was collected on admission. They found out that lactate and cholesterol were the discriminating metabolites predicting 30-day mortality; in particular patient with high lactate and low cholesterol on admission showed increased mortality. This lactate/cholesterol rate in plasma could be a useful and simple parameter to apply in clinical practice in order for the physician to make the best decision in heart failure care.

Another interesting study performed by Deidda et al. in 2015 questioned whether there could be any changes in patients metabolome according to the worsening of their conditions [4]. They compared blood samples of patients affected by mild to moderate impairment of left ventricle ejection fraction and of others affected by severe left ventricle ejection fraction impairment and controls. After the statistical analysis, they identified 3 metabolic clusters related to the 3 groups. The responsible metabolites specific for each heart failure stage are 2-hydroxybutyrate, glycine, methylmalonate, and myoinositol and they might reflect both an increase in energy demand and an impaired ability to generate ATP (see also Table 1). The fingerprint identification of a still-free-of-symptoms myocardial impairment, which directly correlates with the more sensitive echocardiographic parameters of myocardial contractility, could enable a better monitoring of at-risk individuals, allowing the anticipation of systolic function worsening and/or the development of an episode of overt failure.

In line with presented data, a latest review published in the Journal of the American College of Cardiology stated that metabolomics is transforming the ability to predict, identify, and better understand several cardiac diseases, by allowing monitoring of the effectiveness of therapeutic interventions, thus leading to advancing the objective of personalizing the practice of medicine [24].

## 3. Perinatal Programming and Cardiology

The perinatal programming of adult diseases (DOHaD theory) states that every adverse event that may occur during pregnancy “shapes” the health status of the fetus and its development and could affects its life course [25]. Thus this theory emphasizes the importance of this delicate period of life in which everything must be timed properly in order to avoid future complications such as cardiac diseases in adolescence and adult life.

In fact Bassareo et al. investigated the cardiac outcome of young adults born with extremely low birth weight (ELBW) [26]. At 25 years of age they are at risk of major cardiac consequences such as sudden death due to the prolongation in QT interval or they are at higher risk of hypertension due to the reduced brachial-flow mediated vasodilatation compared to those born appropriate for gestational age (see also Table 2) (Bassareo PP, “Long Term Problems in Young Adults Born ELBW” [26–30]).

It is therefore a big challenge, on the opposite side of the life span, to try to predict the cardiac outcome of the neonate during pregnancy. Metabolomics seems to have made it possible even though to our knowledge there is only one study of metabolomics in pregnancy performed by Bahado-Singh et al. [31]. Their aim was to identify metabolomics markers in maternal first-trimester serum for the detection of fetal congenital heart defect. Serum from mothers of CVD fetuses showed a significant disturbance in acylcarnitine and sphingomyelin and other metabolites related to an abnormal lipid metabolism. These findings may help the future development of devices to be used at the bedside similar to those that are already being sold to assess the cardiac troponin in patients with suspected acute coronary syndrome by just a finger prick of blood [32].

Moving from neonates to infants and young adults (since CVDs have a long latent period), metabolomics could be a useful tool to investigate the actual role of genetic predisposition. It could help understand whether a particular gene mutation is protective or harmful and in which metabolism it is involved or if there are any sex differences. A very interesting study concerning this topic was performed by Klein et al. in 2014 [33]. They studied the SORT1 gene through genomics and metabolomics and were able to determine several effects of the mutations of this gene in young males and females.

## 4. Metabolomics Cross Talks with Microbiomics Even in the Occurrence of Heart Diseases?

Metabolomics allows not only measuring changes in metabolites concentrations, but also discriminating those of human origin from those of microbial origins; in fact several authors consider this technology as the Rosetta Stone of microbiomics [34]. Indeed there are 3 types of metabolites in humans: those of human origins (produced only by eukaryotic cells); those of microbial origins (produced only by prokaryotic cells); and those of common origins [35]. Among the most studied ones is hippurate which both is a marker of renal function and can be produced by gut microbiota as well [36]. Nevertheless, in most cases, the origin of the molecules might not be unambiguously determined by using only metabolomics, but this technique is unique in its possibility of simultaneously analyzing molecules from both host and microbes in a single measurement. In a review of 2013 written by Russell et al. they highlighted the different fates of choline for the first time [37]. This metabolite, involved in the methionine-betaine-choline cycle, if metabolized by an altered microbiota, is transformed into trimethylamine N-oxide (TMAO). It is found in several peripheral tissues but in particular in the arterial epithelium in case of atherosclerosis. At the beginning of this year, a very interesting study was performed by Feng et al. in which the integrated metabolomics and metagenomics analysis of plasma and urine allowed identifying metabolites of microbial origins in coronary heart disease [7]. These molecules, mannitol and N-acetyl-D-glucosamine-6-phosphate among others, are related to a particular strand of* Clostridium *spp. or* Streptococcus *spp., indicating a possible role of the dysbiosis in the occurrence of this pathology. In November 2016 a review written by Jonsson and Bäckhed was published, concerning all the recent literature about the role of gut microbiota in atherosclerosis [38]. They stated that specific strand of bacteria, Proteobacteria, can be found in the atherosclerotic plaque. This phylum comprises the genera of* Helicobacter* and* Chryseomonas *and it is the most abundant in the plaque. Actinobacteria can be found in the plaque as well, while in patients with coronary artery disease (CAD) the number of Bacteroidetes decreases compared to controls and the ratio of Firmicutes/Bacteroidetes increases. They also proposed 3 possible mechanisms by which microbiota could affect the development of atherosclerotic plaque.

The first one: bacterial infection activates the immune system causing an excessive inflammatory response that may turn out to be dangerous, independently of the site of invasion. The subsequent proatherogenic response could be mediated by Toll-like receptor 4 expressed in macrophages.

The second: the TMAO production could initiate the activation of platelets and foam cells.

The third: the production of noxious molecules such as the previously mentioned TMAO is related to the diet and gut microbiota metabolites.

With metabolomics it is possible to demonstrate that apparently healthy young adults who were born with birth weight < 1000 g present a specific profile compared to apparently healthy young adults who were born at term. That is perinatal programming.

Differences in the two groups were related to the alterations in the arginine and proline metabolism, in the purine and pyrimidine metabolism in the histidine, in beta-alanine metabolism, and in the urea cycle [39].

## 5. Future Perspectives

The most investigated cardiac pathologies are heart failure, coronary heart disease, and myocardial infarction.

Several studies displayed an alteration of metabolites concerning lipid metabolism, highlighting the energy imbalance as a peculiar feature of such pathologies.

On the other hand, some authors showed different metabolites that indicate an interaction between diet and microbiota.

These findings open up unusual scenarios to the cardiologist and although it is normal to feel some sort of incredulity, they could pave the way to new possibilities of early diagnosis and individualized treatment. Congenital malformations, gut colonization by microbiota, individual genetic arrangement, and its interplay with both behavioral and risk factors, such as drugs assumption, can influence the occurrence of heart diseases. Metabolomics, for its peculiarities, seems to be the most promising technology to investigate the individual predisposition or the eventual long-term prognosis of these pathologies.

## Figures and Tables

**Table 1:** Recent relevant works concerning metabolomics in cardiology are shown chronologically with the main metabolites shifts.

Authors	Patients	Methods	Sample	Metabolites results
Feng et al. 2016 [7]	59 CHD patients and 43 healthy controls	Untargeted metabolomics method	Plasma, urine	↑ GlcNAc-6-P and mannitol in CHD
Ahmad et al. 2016 [8]	41 patients with end-stage heart failure	Tandem flow injection Mass spectrometry	Plasma	↑ long chain acetylcarnitines in chronic heart failure
Oni-Orisan et al. 2016 [9]	123 patients with coronary artery disease (CAD) versus 39 controls	Mass spectrometry	Plasma	↓ cytochrome P450-derived epoxyeicosatrienoic acids metabolites in CAD
Deidda et al. 2015 [4]	24 heart failure patients versus 9 controls	^1^H-NMR	Plasma	↓ 2-hydroxybutyrate, in HF patients ↑ glycine, methylmalonate, and myoinositol in HF patients
Zordoky et al. 2015 [10]	44 HF patients versus 20 controls	LC/MS ^1^H-NMR	Serum	↑ acylcarnitines, carnitine, creatinine, betaine amino acids, ketone bodies in HF patients ↓ phosphatidylcholines, lysophosphatidylcholines, sphingomyelin in HF patients
Cheng et al. 2015 [11]	401 HF patients versus 114 controls	Mass spectrometry	Plasma	↓ phosphatidylcholines, arginine ↑ ornithine, spermidine, spermine phenylalanine, tyrosine
Würtz et al. 2015 [12]	1373 cardiovascular events	Quantitative nuclear magnetic resonance	Serum	↑ phenylalanine, monounsaturated fatty acids in cardiovascular events ↓ omega 6 fatty acids, docosahexaenoic acids in cardiovascular events
Zhong et al. 2014 [13]	157 hypertension patients versus 99 controls	^1^H-NMR	Serum	↑ VLDL, LDL, lactic acid, acetone ↓ valine, alanine, pyroacemic acid, inose, p-hydroxyphenylalanine, methylhistidine in hypertension patients
Vaarhorst et al. 2014 [14]	79 cases of coronary heart disease	^1^H-NMR	Plasma and serum	↑ ornithine, TMAO ↓ valine, arginine, creatinine
Shi et al. 2014 [15]	45 cases of coronary heart disease versus 15 controls	^1^H-NMR	Plasma	↑ leucine, N-acetyl glycoprotein, *α*-glucose, *β*-glucose, phenylalanine, acetone, HDL, glutamate, glutamine, methylalanine, lysine, tyrosine, ornithine, taurine, proline, lactic acid, tryptophan, valine, acetyl-glutamic acid ↓*β*-hydroxy-isobutyric acid
Rizza et al. 2014 [16]	17 major cardiovascular events (MACE) patients versus 50 controls	Mass spectrometry	Serum	↑ medium and long chain acylcarnitines in MACE patients
Kalim et al. 2013 [17]	100 individuals dead of a cardiovascular cause versus 100 controls	Liquid chromatography/mass spectrometry	Plasma	↑ oleoyl carnitine in CV patients
Tenori et al. 2013 [18]	185 heart failure patients versus 111 controls	^1^H-NMR	Serum, urine	↑ phenylalanine, tyrosine, isoleucine, creatine, TMAO, lipid, formate, lipoprotein, hypoxanthine, proline, urea, dimethylamine, serine, acetate, methanol ↓ valine, choline, arginine, creatinine, dimethylsulfone, Gln+ Gli, alanine, l-dopa, dimethylglycine, citrate, lactate, lysine, uridine, methionine
Desmoulin et al. 2013 [19]	126 acute heart failure (AHF) patients	^1^H-NMR	Plasma	↑ lactate + ↓ low cholesterol = ↑ short term mortality
Samara et al. 2013 [20]	25 acute decompensated heart failure (ADHF) patients versus 16 controls	Selected ion flow tube mass spectrometry	Breath	↑ acetone, pentane in ADHF
Magnusson et al. 2013 [21]	253 cardiovascular disease patients (CVD) versus 253 controls	Liquid chromatography/mass spectrometry	Plasma	↑ branched and aromatic amino acids in CVD
Bodi et al. 2012 [22]	20 angioplasty induced myocardial ischemia versus 9 controls	^1^H-NMR	Serum	↑ phosphoethanolamine, lactate, glucose, tyrosine, phenylalanine, glycerol in AIMI
Kang et al. 2011 [23]	15 heart failure patients versus 20 controls	^1^H-NMR	Urine	↑ acetate, acetone, methylmalonic acid, cytosine, phenylacetylglycine ↓ 1-methylnicotinamide

**Table 2:** Possible long-term consequences in adulthood to subjects born with extremely low birth weight: suggestion for diagnosis and care.

Possible consequences in adulthood	Risks	Suggestion for diagnosis and care
Increase in the QT interval of ECG in some subjects	Risk of arrhythmia and sudden death	ECG monitoring Avoidance of drugs that increase the QT interval
Reduced vascular elasticity	Risk of hypertension	Blood pressure monitoring
High ADMA levels	Risk of acute cardiovascular problems	ECG and blood pressure monitoring
Increase in microalbuminuria and urinary NGAL, reduction of kidney volume	Risk of chronic kidney insufficiency	Urine stick monitoring, albuminuria, creatinine, and cystatin C in the blood, kidney ultrasound
